# Cultural adaptation and validation of the Portuguese version of the Nursing Clinical Facilitators Questionnaire

**DOI:** 10.1590/1518-8345.0617.2767

**Published:** 2016-08-15

**Authors:** Maria Manuela Frederico-Ferreira, Ana Paula Forte Camarneiro, Cândida Rosalinda Exposto da Costa Loureiro, Maria Clara Amado Apóstolo Ventura

**Affiliations:** 1 PhD, Assistant Professor, Escola Superior de Enfermagem de Coimbra, Coimbra, Portugal.; 2 PhD, Adjunct Professor, Escola Superior de Enfermagem de Coimbra, Coimbra, Portugal.

**Keywords:** Mentors, Nursing Education Research, Education, Nursing

## Abstract

**Objective:**

to perform the cultural adaptation to Portuguese of the Nursing Clinical Facilitators Questionnaire (NCFQ), which was designed by the Centre for Learning and Teaching at the University of Technology of Sydney, and to validate this instrument.

**Methods:**

this methodological study involved the cultural adaptation of the questionnaire by using translation, back-translation, semantic comparison, idiomatic and conceptual equivalence, and validation through validity and reliability analyses and used a sample of 767 students in their second year of the Nursing Program.

**Results:**

construct validity had a two-factor solution according to the varimax rotation method. In addition, there was a high overall internal consistency for the questionnaire (Cronbach's alpha of 0.977) and for the factors found (0.966 and 0.952, respectively).

**Conclusion:**

the Portuguese version has good psychometric characteristics; therefore, it is adequate to obtain reliable information on the perception of nursing students concerning the type of supervision that is provided in clinical practice, and this version is adequate to improve teaching practices.

## Introduction

The monitoring of nursing students in clinical teaching (CT) is demanding for all involved. The CT program of the Graduate Studies in Nursing (GSN) has become law in Portugal since September 18, 1999 by Ordinance No. 799-D/99^1^. Article 3 of this law states that "clinical teaching is ensured via internships and should be conducted in health facilities and in the community under the supervision of University teachers, with the assistance of skilled health personnel."

The study plan of the GSN from the Nursing School of Coimbra (Escola Superior de Enfermagem de Coimbra-ESEnfC), Portugal, follows the European Credit Transfer System (ECTS). It lasts four years/eight semesters and requires 240 credits that are distributed in the areas of Theory (T), Theory and Practice (TP), Laboratory Practice (LP), and Clinical Teaching (CT). The practical component constitutes approximately 50% of the total credits and includes CT, which is considered, at the European level, "that part of nurse training in which trainee nurses learn, as part of a team and in direct contact with a healthy or sick individual and/or community, to organise, dispense and evaluate the required comprehensive nursing care, on the basis of the knowledge and skills which they have acquired"^2^. 

When considering the number of credits that are allocated to CT in the GSN, its importance is evident. CT is regarded as a significant opportunity to confront students with actual work situations. In addition, CT contextualizes and consolidates theoretical knowledge and constructs new knowledge and learning through critical reflection, creative thinking, and problem-solving^3^. Its main objective is the development of personal, psychosocial, clinical, ethical, and deontological skills.

Nursing Fundamentals is the first hospital-based CT in ESEnfC and is taught in the second year. It is supervised by guest assistants and comprises active nurses, who, in turn, are supervised by the university's professors. The workload that is established for supervising students is 8 hours per day, 35 hours per week, per student group. The group has 4-6 students and at least two supervisors in each CT. In the other CTs, student supervision is ensured primarily by the faculty of the School.

The epistemological value of this practice, which was advocated by Schӧn, is that the practice of CT requires a practical rationality that is based on knowledge or supervised practice, which is opposed, somewhat, to the basis of the traditional culture of higher education teachers that is epistemologically sustained by a positivist root called technical rationality^4^. In these circumstances, the following three fundamental aspects should be considered: an investment in teaching in the clinical setting and the definition of strategies that connect theory and practice to facilitate context-based learning, which operates in pedagogical mediation from a perspective of decentralization, symmetry, and reciprocity^5^ ^-^ ^6^; the appropriate intellectual, professional, and personal development of CT supervisors as a prerequisite to CT, which is considered as a network of interacting forces that influence student learning^5^ ^-^ ^7^; and the monitoring of the satisfaction of students with the supervisors in the successive transformations of experience.

Therefore, the Ministerial Order No. 1/87 from April 21, 1987, which was reinforced by Decision No. 8/90 from February 28, 1990^8^, is in force. This Order emphasizes that teachers should supervise and evaluate the practical learning of students with the help of service personnel. Therefore, the teacher should not only transmit knowledge and information but also teach and empower students to articulate this knowledge and use it in practice^9^. It is essential to promote students' ability to think, analyze, and seek explanations in different learning situations^6^ ^-^ ^10^, including (1) professional skills in theoretical and practical teaching, assessment knowledge, and good interpersonal relationships and (2) the encouragement of critical thinking and the ability to teach how to integrate theoretical knowledge with nursing practice. Moreover, the teacher should be able to manage and help others to manage new strategies for the conciliation of new paths of discovery and the unexpected, which requires an increased allocation of time, understanding, and care from all who are involved in the complexity of CT^5^. 

The mobilization of the knowledge and the replacement of individual thought for an interconnection of all knowledge will facilitate the resolution of situations that arise; in addition, in education, what students learn, not what teachers teach, is essential^11^.

The training of a nurse is deeply dependent on the quality of learning in the clinical setting. Training in this complex interface that involves teaching, learning, and personal transformation facilitates the psychological process of adherence to work activities and allows the reflexive rationalization of the various dimensions of health problems. This training also provides the basis for the establishment of self-consciousness as a future professional in a game of adjustment of roles and is crucial to social and moral development through a combination of logics in the students' entire dimension^5^.

The skill-based model of education that is used in CT at ESEnfC allows teachers and supervisors to become facilitators of learning and allows students to become the constructors of their own knowledge. Students are taught that "learning to learn" should be a voluntary and autonomous process in the construction of knowledge and learning; therefore, they must become responsible for their education. However, the implementation of this model faces some difficulties, including the choice of the most suitable strategies for acquiring specific knowledge and expertise. In this context, not all students respond equally in terms of motivation and learning, particularly in large groups. Therefore, students should understand their role and the role of teachers and institutions^12^ in ensuring the quality of the teaching and learning processes.

To ensure the quality of the assessment that is made by the education community, ESEnfC students are asked to comment on their satisfaction with the supervision in CT, although some teachers consider student evaluations as unfair and unacceptable^13^. Student opinions on the effect of teaching practices in CT help supervisors to reflect on the message that is taught to students and is similar to the feedback that is received by teachers in class^14^. The Council for Quality and Evaluation (Conselho para a Qualidade e Avaliação-CQA) is responsible for this assessment, which is defined by statute. The processes are monitored by using procedures that are designed to measure what is done, how it is done, and the degree of satisfaction of the people who are involved in the teaching and learning processes. The results of this evaluation allow the implementation of improvements that are necessary to the development of a quality policy that is increasingly based on assessments, autonomy, and responsibility and with the participation of all involved elements. 

Concerning the supervision of CT by guest assistants, the school should know the students' degree of satisfaction with the supervision that is provided. We reviewed the literature on nursing students' opinions of CT supervision by using all EBSCO databases and the descriptors of 'student evaluation of teaching' and 'clinical learning environment'. We selected the Nursing Clinical Facilitator Questionnaire (NCFQ), which was created by the Centre for Learning and Teaching at the University of Technology in Sydney, Australia^15^. This questionnaire has been used in different cultural contexts and has shown good psychometric properties in evaluating the satisfaction of nursing students with their supervision in CT. The original version was published by Espeland and Indrehus^15^ and Raholm, Thorkildsen, and Lofmark^16^ and adapted to a sample of Norwegian students. The questionnaire contains 27 statements, and each statement has five possible answers on a Likert scale. The questionnaire is based on the following three factors: 1: learning-supporting behavior (items 7, 9, 17, 18, 19, 20, 21, 22, 24, and 25); 2: learning-stimulating behavior (items 5, 6, 10, 11, 12, 13, 14, 15, 16, and 23); and 3: preparatory behavior (items 1 and 2). 

The objectives of this study were to perform the cultural adaptation to Portuguese of the Nursing Clinical Facilitators Questionnaire (NCFQ) and to validate the instrument.

## Methods

For the creation of the Portuguese version of the Nursing Clinical Facilitator Questionnaire (NCFQ), a methodological study of cultural adaptation and validation was conducted. Although this instrument has been published and has no restrictions on its use, we petitioned for an authorization to use this instrument, but the authors never replied. Therefore, we initiated the process of cultural and linguistic adaptation and searched for similar instruments that were written in languages other than the original, including translation, back-translation, semantic comparison, and idiomatic and conceptual equivalence^17^. We ensured that the translations yielded results with similar interpretations, including the results concerning semantics and contents^18^.

In the questionnaire translation stage, we intended to obtain a version in Portuguese that was linguistically correct and equivalent to the original version. This stage began with the creation of two Portuguese versions and was performed independently by two translators who were native in the Portuguese language and fluent in English. This step was followed by the stage of reconciliation between the two translations, and their contents were analyzed and compared. This phase included the presence of the two translators and two study researchers. The identified discrepancies were minimal and related solely to the words that can have different translations in Portuguese. Therefore, the first Portuguese version was obtained and was subjected to back-translation by a native English-speaking translator. Later, the researchers compared this version with the original version. This stage was followed by the submission of the project to the Ethics Committee (Opinion No. 224-09/2014).

In a later phase, we asked a group of five nursing professors, who were experts in clinical supervision, to make a critical assessment of the content of the questionnaire concerning its technical, linguistic, and semantic aspects^19^ as well as an assessment of the clarity and relevance of each item and the adequacy and suitability of this instrument to achieve the proposed goals^20^. The agreement rate was higher than 95%.

The next stage was the pilot test, which involved the completion of the questionnaire by a group of 21 students; in addition, these students were asked to evaluate the content of the questionnaire items to assess their clarity and adequacy. Later, the questionnaire was discussed. There were no difficulties in understanding or ambiguities in the interpretation of the questionnaire. 

Considering the need to obtain more detailed responses for the evaluated items and to make them consistent with the responses of other ongoing studies, in the Portuguese version, we decided to include seven possible answers to each item (which ranged from 1 to 7, where 1 indicated "strongly disagree" and 7 indicated "strongly agree"). The obtainment of a Portuguese version was followed by the development of a set of reliability and validity tests by using international statistical standards. Regarding validity, a value of 0.50 was accepted as the minimum factor loading for each scale item. 

The questionnaire was applied to a convenience sample of 767 students from the second year of the GSN program of the ESEnfC, who were enrolled in hospital-based CT in Coimbra, Portugal, in the academic year of 2013/2014. The inclusion criteria were being a second-year student and having completed one of the CT modules on the fundamentals of hospital-based nursing. Each student completed a questionnaire concerning each supervisor. The questionnaires were voluntarily self-completed by using an electronic platform, and participant anonymity was ensured. The data were processed by using IBM SPSS software version 22.

## Results

The assessment of the validity and reliability of the questionnaire revealed the following results. To analyze construct validity, a factorial analysis was conducted by using principal component analysis followed by orthogonal varimax rotation. According to the original version^16^, the last two global statements (items 26 and 27) that summarize the classification of supervisors as CT teachers and the learning of clinical practice were not included in the exploratory factor analysis.

Subsequently, the Kaiser-Meyer-Olkin (KMO) measure was determined, and Bartlett's test of sphericity was conducted. Solutions with eigenvalues greater than 1.00 were found. The KMO measure was 0.976, which allowed the performance of a factor analysis. The value of Bartlett's test of sphericity was χ 2 = 19013.04 at p <0.001, where H0 was rejected, and we concluded that the variables were significantly correlated. The initial commonalities were 1, and for the extracted factors, the percentage of variance of each explained variable was greater than 0.50. By using the rule of retention for the factors with eigenvalues that were ​​greater than 1.00, which was confirmed by the Scree plot, two factors that explained 69.86% of the total variance were extracted. The first factor explained 37.180% of the variance, and the second factor explained 32.686% of the variance. Therefore, the factorial solution that was found was favorable from statistical and significance viewpoints.

Factor I consisted of 13 items, and factor II consisted of 12 items. No items were eliminated. Item 8, 'The supervisor encourages me to be responsible for my learning', predominates in the two factors, which indicates that learning can be explained by the support and stimulation of learning. We maintained the item in the dimension where its factor weight was higher. 

Table 1 shows the factor weights of each item of the two factors that were obtained from the students' opinions on their supervision in CT and the percentage of explained variance after exploratory factor analysis and orthogonal varimax rotation (n = 767).

**Table 1:** Factor weight of each item of the Nursing Clinical Facilitators Questionnaire that was administered in Coimbra, Portugal, in the academic year of 2013/2014 and the percentage of the explained variance that was obtained in the principal component analysis with varimax rotation.

Questionnaire items	Factor I	Factor II	Commonality
4. The supervisor is aware of my level of previous learning and competence	0.601		0.629
5. The supervisor discusses my learning needs with me	0.586		0.673
6. The supervisor gives me a clear idea of ​​what is expected of me in clinical practice	0.651		0.639
7. The supervisor gives me enough learning opportunities for my independence in clinical practice	0.649		0.669
9. The supervisor provides an appropriate amount of support for my level of experience	0.656		0.781
17. The supervisor gives me enough feedback about my progress	0.867		0.822
18. The supervisor gives me enough feedback to help me improve	0.862		0.863
19. The supervisor's feedback is honest	0.759		0.673
20. The supervisor provides feedback at appropriate times during and/or after clinical practice	0.780		0.754
21. The supervisor shows interest in my learning	0.765		0.783
22. The supervisor is open to the opinion of others	0.659		0.647
23. The supervisor encourages students to get the maximum benefit from sharing learning experiences	0.679		0.749
25. The supervisor is approachable	0.685		0.718
8. The supervisor encourages me to be responsible for my learning	0.573	0.615	0.707
1. The supervisor ensures that clinical practice is organized in advance with the nursing staff		0.660	0.575
2. The supervisor ensures that users agree with the participation of students in clinical practice		0.711	0.534
3. The supervisor discusses with me his/her availability to advise me		0.596	0.668
10. The supervisor informs me when I should intervene to maintain user safety and comfort		0.643	0.684
11. The supervisor helps me link theory with clinical practice		0.751	0.705
12. The supervisor makes me aware of the legal implications of treatment decisions		0.747	0.664
13. The supervisor encourages me to consider a range of alternative methods of user care		0.723	0.720
14. The supervisor makes me aware of aspects of clinical situations to increase my existing knowledge		0.732	0.761
15. The supervisor motivates me to reflect on my clinical learning		0.598	0.680
16. The supervisor shows me how to make decisions about user care		0.641	0.760
24. The supervisor seems confident in his/her role as a clinical teacher		0.588	0.610
Percentage variance explained	37.180	32.686	
Cumulative percentage variance explained	37.180	69.866	

Despite slight differences in some items relative to the original proposal, the representation of the results that were obtained allowed maintaining the identical names for the factors after analyzing the number and content of the items. Factor 1 (items 4, 5, 6, 7, 9, 17, 18, 19, 20, 21, 22, 23, and 25) was designated "learning-supporting behavior" and factor 2 (items 1, 2 3, 8, 10, 11, 12, 13, 14, 15, 16, and 24) was designated "learning-stimulating behavior". 

In the original version, factor 1 (items 7, 9, 17, 18, 19, 20, 21, 22, 24, and 25) was designated "supportive behavior", and factor 2 (items 5, 6, 10, 11, 12, 13 14, 15, 16, and 23) was designated "challenging behavior". 

Internal consistency was determined to assess reliability by calculating Cronbach's alpha, which is one of the most commonly used coefficients to evaluate this variable in Likert scales^18^. The corrected item-total correlation was also determined. We observed that the overall Cronbach's alpha of the questionnaire was 0.977 and that the lowest value of the item-scale correlation was 0.583, i.e., higher than 0.50, which agrees with the value that was determined by the authors of the version under study to accept the item on the scale. Alpha was 0.966 in factor I and 0.952 in factor II.

## Discussion 

The adaptation of the NCFQ to Portuguese proved to be very useful because given its psychometric properties, this instrument fills an assessment gap of the staff of the Council for Quality and Evaluation that is the same as the objective of the questionnaire, namely, to measure the degree of satisfaction of students with the supervision that they received during CT^15^.

No difficulties in the process of cultural adaptation were detected; on the contrary, a text as close as possible to the original was obtained, and no semantic changes were required.

Regarding validation, the questionnaire showed a strong internal consistency (Cronbach's alpha of 0.977), which was higher than that found by the authors. Factor analysis with varimax rotation indicated the presence of two factors that were very similar to the factors in the original version. However, the original version had a third factor with two items (1 and 2), which were designated preparatory behavior; however, these items were not found in this study. Furthermore, in the original study, three items (3, 4, and 8) had the same weight in the two factors; the weight of these factors was lower than 0.50, and therefore, they were removed by the authors. In this study, this weight was not found, except for item 8, and the items were maintained.

Therefore, these factors represented learning-supporting and -stimulating behaviors and had a high internal consistency (0.966 and 0.952, respectively); however, these values were higher than the values that were found in the study by Espeland and Indrehus^15^.

The first factor contained items such as "My supervisor discusses my learning needs with me", "My supervisor gives me a clear idea of ​​what is expected of me in clinical practice", and "My supervisor gives me enough learning opportunities for my independence in clinical practice." These items help to identify strategies that facilitate contextual learning, i.e., learning that involves theory and practice^5^ and is based on knowledge or guided practice^4^.

The second factor has items such as "My supervisor helps me link theory with clinical practice", "My supervisor encourages me to consider a range of alternative methods of user care", and "My supervisor encourages me to reflect on my clinical learning". These items promote the ability to think, analyze, and seek explanations in different learning situations^10^, which demonstrates the intellectual development of the students^5^.

The small differences in the distribution of the items in the factors compared with the scale that was used by Espeland and Indrehus^15^ in Norway can relate to cultural, linguistic, and interpretive differences. For this reason, these differences were accepted. 

## Conclusion

The evaluation of the psychometric properties of the questionnaire indicated that they are similar to those of the translated version; thus, the questionnaire is adequate to assess the degree of satisfaction of students with their supervision in CT.

Therefore, this instrument allows the development of future studies on this topic and the comparison with the results that are obtained in other countries where this instrument has been or will be used.

We believe that this validation increases the number of valid instruments to assess students' satisfaction with their supervision in CT. Our research team will perform confirmatory factor analysis in the near future.
